# Bootstrap-after-Bootstrap Model Averaging for Reducing Model Uncertainty in Model Selection for Air Pollution Mortality Studies

**DOI:** 10.1289/ehp.0901007

**Published:** 2009-09-17

**Authors:** Steven Roberts, Michael A. Martin

**Affiliations:** School of Finance and Applied Statistics, College of Business and Economics, Australian National University, Canberra, Australian Capital Territory, Australia

**Keywords:** air pollution, Bayesian, bootstrap, model averaging, mortality, particulate matter

## Abstract

**Background:**

Concerns have been raised about findings of associations between particulate matter (PM) air pollution and mortality that have been based on a single “best” model arising from a model selection procedure, because such a strategy may ignore model uncertainty inherently involved in searching through a set of candidate models to find the best model. Model averaging has been proposed as a method of allowing for model uncertainty in this context.

**Objectives:**

To propose an extension (double BOOT) to a previously described bootstrap model-averaging procedure (BOOT) for use in time series studies of the association between PM and mortality. We compared double BOOT and BOOT with Bayesian model averaging (BMA) and a standard method of model selection [standard Akaike’s information criterion (AIC)].

**Method:**

Actual time series data from the United States are used to conduct a simulation study to compare and contrast the performance of double BOOT, BOOT, BMA, and standard AIC.

**Results:**

Double BOOT produced estimates of the effect of PM on mortality that have had smaller root mean squared error than did those produced by BOOT, BMA, and standard AIC. This performance boost resulted from estimates produced by double BOOT having smaller variance than those produced by BOOTand BMA.

**Conclusions:**

Double BOOT is a viable alternative to BOOT and BMA for producing estimates of the mortality effect of PM.

Over the past decade, time series studies that have investigated the association between daily variations in particulate matter (PM) air pollution and daily variations in mortality have become commonplace (Breitner et al. 2009; Kelsall et al. 1997; Roberts 2004). Studies conducted in Europe and North America have found statistically significant associations between increases in daily PM concentrations and increases in daily mortality (Samoli et al. 2008). One common feature of these time series studies is that myriad modeling choices must be made to arrive at an “optimal” model from which an estimate of the association between PM and mortality can be obtained. This array of choices means there are potentially many candidate models for investigating the association between daily PM and mortality. In some studies, models that are selected because they optimize a particular model selection criterion are used to infer a relationship between PM and mortality (Draper 1995; Goldberg et al. 2006; Kelsall et al. 1997). In this context, concerns have been raised in the literature about statistical issues that may arise from the process of selecting a single model from among a potentially large number of competing candidates (Clyde 2000; Koop and Tole 2004; National Research Council 1998). The procedure of selecting a single “best” model may ignore the model uncertainty, which is inherently involved in searching through the set of candidate models to determine the best one. Ignoring model uncertainty is problematic because it reflects statistical variation not captured within the single chosen model, and failure to account for this variation can increase the chance of erroneously concluding a statistically significant association between PM and mortality (Clyde 2000; National Research Council 1998).

Model averaging in both Bayesian and frequentist forms has been proposed as a means of allowing for model uncertainty in time series studies of PM and mortality (Clyde 2000; Koop and Tole 2004, 2006; Martin and Roberts 2006). Model-averaging procedures assign probabilities or weights to each candidate model that reflect the degree to which the model is supported by the data. These probabilities can be used to produce “weighted” average estimates of the association between PM and mortality that explicitly incorporate information from each candidate model. This process of explicitly incorporating each candidate model into the estimation process produces estimates that incorporate the variation inherent in the model selection process. Clyde (2000) and Koop and Tole (2004, 2006) implemented Bayesian model-averaging (BMA) techniques to estimate the association between air pollution and mortality. Martin and Roberts (2006) implemented model averaging using a bootstrap-based procedure and showed that it is competitive with BMA in that context. Previous investigations have also used the bootstrap in the context of time series studies of air pollution, including investigations of the effect of concurvity in generalized additive models (Figueiras et al. 2005; Ramsay et al. 2003).

In this paper, we discuss a double bootstrap model-averaging (double BOOT) approach that extends and improves the bootstrap model-averaging (BOOT) procedure that was implemented in Martin and Roberts (2006).

## Materials and Methods

### Materials

The data used in this report were obtained from the publicly available National Morbidity, Mortality, and Air Pollution Study database (Zeger et al. 2006). The data consist of daily time series of mortality, temperature, dew point temperature, and PM air pollution measures for five United States (U.S.) cities for the period 1999–2000. The mortality data are daily counts of nonaccidental deaths of individuals ≥ 65 years of age. The measure of ambient PM used is the ambient 24-hr concentration of PM of < 2.5 μm in aerodynamic diameter (PM_2.5_) measured in micrograms per cubic meter.

The five U.S. cities included in this study—Birmingham, Alabama; Orlando, Florida; Seattle, Washington; St. Louis, Missouri; and Tampa, Florida—were selected because they had nearly complete PM_2.5_ data over the period of investigation. For these cities, the number of days missing PM_2.5_ concentrations over the 730-day period of investigation ranged from 2 to 18 days. Missing PM_2.5_ concentrations were imputed using the average of the previous and subsequent days’ concentrations.

### Methods

We investigated model averaging in the context of additive Poisson log-linear models. Under these models, the daily mortality counts are modeled as independent Poisson random variables with mean μ*_t_* on day *t*


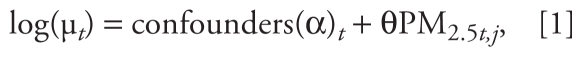


where confounders(α)*_t_* represent other time-varying variables related to daily mortality, and PM_2.5_*_t_*_,_*_j_* is the PM_2.5_ concentration on day *t*–*j*, for a specific time lag *j*; α is a tuning parameter—as α increases, so too does the flexibility of the smooth functions used to adjust for the effects of the confounders. Adjusting for confounders is important to avoid spurious findings of an association between PM_2.5_ and mortality (Bell et al. 2004). Commonly used confounders include weather variables, such as temperature and dew point temperature, and time (Dominici et al. 2003). Our focus in Model [1] on a PM_2.5_ exposure measure, which corresponds to a specific lag of PM_2.5,_ is consistent with recent time series studies (Dominici et al. 2006, 2007; Peng et al. 2009). Models of the same general form as Model [1] are commonly used in time series studies of the adverse health effects of PM (Dominici et al. 2007; Peng et al. 2006; Roberts 2005).

Using Model [1] involves selecting a value of α and a lag of PM_2.5_. For example, if *p* values of α and *q* lags of PM_2.5_ were thought plausible, then *K* = *p* × *q* candidate models could be fitted and assessed with respect to some model selection criterion. If the *K* candidate models are fitted and a single “best” model chosen, the common practice of reporting the statistical characteristics of the winning model effectively ignores the statistical variation suffered as a result of the model selection procedure itself.

In the paragraphs that follow, we describe Akaike’s information criterion (AIC; Akaike 1973) and outline the bootstrap (BOOT) method used by Martin and Roberts (2006) and our extension that refines this method.

AIC is commonly used for model selection in time series studies of the association between PM and mortality (Goldberg et al. 2006; Samoli et al. 2008). It takes a measure of the lack of fit of a model and adds a penalty for the number of parameters in the model. Specifically, AIC is defined as


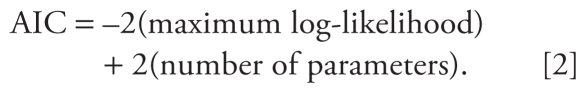


To use AIC for model selection, the model with the smallest value of AIC among the candidate models is selected. Further details on AIC, including a discussion of its derivation, can be found in numerous articles (e.g., Burnham and Anderson 2004). In the context of the models considered in this paper, the number of parameters is an increasing function of α.

The BOOT method used by Martin and Roberts (2006) proceeds through the following steps:

1. Fit the *K* candidate models defined by Model [1]. Select as “best” the model with the smallest value of AIC, which is denoted M*. We also define *M_i_* to represent candidate model *i* fitted to the observed mortality time series data, for *i* = 1, 2,…, *K*.
2. Extract the mean adjusted, standardized Pearson residuals (Davison and Hinkley 1997a) and the estimated mean mortality counts from the best model M*, which was obtained in step 1. In our context, the mean adjusted, standardized Pearson residuals ζ*_t_* are defined as

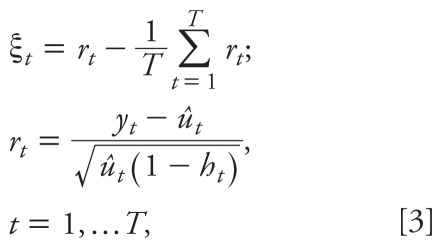
  where *T* is the length of the mortality time series, *y_t_* is the observed mortality count on day *t*, *û_t_* is the estimated mean mortality count on day *t*, and *h_t_* is the leverage for the observation on day *t*.
3. Use the stationary bootstrap to generate *B* resamples of the residuals ζ_1_,…, ζ*_T_* obtained in step 2. The stationary bootstrap is implemented using the approach of Politis and Romano (1994). Under this approach, the stationary bootstrap resamples blocks of data of random length, where the length of each block has a geometric distribution.
4. Create *B* bootstrap replicate mortality time series by adding the estimated mean mortality counts from step 2 to each of the *B* resampled residual series generated in step 3. This process is carried out using the following formula:

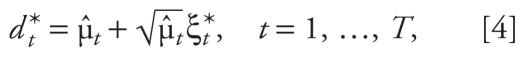
  where ζ_1_*,…, ζ_T_* is a resampled residual series, and *d*_1_*,…, d_T_* is the resultant bootstrap replicate mortality time series. The *d*_1_*,…, d_T_* are rounded to the nearest integer before proceeding to step 5.
5. Using each of the *B* bootstrap replicate mortality time series, repeat step 1 with the observed mortality time series data replaced by the bootstrap replicate mortality time series, each time tabulating which of the *K* models is “best” based on AIC.
6. Assign a weight *w_i_* equal to the proportion of the *B* times that the model was selected as best in step 5, to each of the *K* candidate models.
7. Use the weights obtained in step 6 to compute a “bootstrap weighted” estimate for the effect of PM_2.5_ on mortality: *w*_1_θ̂_1_+…+ *w_K_*θ̂*_K_*, where θ̂*_i_* is the estimated effect of PM_2.5_ on mortality obtained from *M_i_*.

In step 3, the stationary bootstrap is used to allow the resampled residuals to mimic the dependence structure of the original residual process under the notion that, although adjacent data points might suffer dependence, blocks of sufficient length may be close to independent of one another. Based on our earlier work, the stationary bootstrap is implemented using a mean block length of size 10 (Martin and Roberts 2006). Lahiri (2003) provides additional information on the use of resampling methods for dependent data. It is important to note that the replicate mortality time series generated in step 4 are not Poisson distributed, but this issue is not of particular concern because the observed mortality time series will also not be Poisson distributed. Indeed, some studies explicitly allow for the non-Poisson nature of the observed mortality time series via quasi-likelihood estimation (Goldberg et al. 2006; Samoli et al. 2008). In our context, the overdispersion estimated within the framework of a Poisson generalized linear model was mild. Thus, we did not consider a quasi-likelihood approach necessary. Further information on residual-based resampling for generalized linear models can be found in Davison and Hinkley (1997b).

Our extension to BOOT described above (termed “double BOOT”) uses a second bootstrap layer after step 6. The second bootstrap layer involves generating another *B* bootstrap replicate mortality time series that are based on the weights *w_i_* found for each model in the first bootstrap layer. For each of the *K* candidate models, this procedure involves generating *Bw_i_* replicate mortality time series using model *M_i_* as the basis for the bootstrap procedure described above, for each *i* = 1, 2,…, *K*. As before, based on this new set of *B* replicate mortality time series, updated weights are constructed for each model based on the proportion of times it was selected as best based on AIC.

The procedure for implementing double BOOT is as follows:

1. Perform steps 1–6 above of the BOOT method.
2. For each of the *i* = 1 to *K* candidate models, construct *Bw_i_* replicate mortality time series using the procedure described in steps 2–4 of BOOT with M* replaced by *M_i_*. This process will produce *B* = *Bw*_1_ +…+ *Bw_K_* second-layer bootstrap replicate mortality time series.
3. Fit the *K* candidate models to each of the *B* replicate mortality time series, each time noting which of the *K* models is “best” based on AIC.
4. Assign a weight w_i_* to each of the *K* candidate models. For each model, the weights are calculated as the proportion of the *B* times the model was selected as best in the preceding step.
5. Use the weights w_i_* to compute a double-bootstrap weighted estimate for the effect of PM_2.5_ on mortality: *w*_1_*θ̂*_1_* +…+ w_K_* θ̂*_K_*, where θ̂*_i_* is the estimated effect of PM_2.5_ on mortality obtained from *M_i_*.

A rationale for this proposed extension to BOOT can be provided through a simple example. Consider a setting where there are only two candidate models, and model 1 is judged as best based on AIC. Now suppose the original BOOT procedure is implemented resulting in weights of *w*_1_ = 0.51 and *w*_2_ = 0.49 being assigned to models 1 and 2, respectively. The original BOOT procedure simply uses these weights to produce an average effect estimate. However, the weights of 0.51 and 0.49 can be interpreted as the data providing essentially equal support for the two candidate models. This outcome poses the question of whether it is desirable for the bootstrap replicate mortality time series to be constructed solely on the basis of model 1 when, in fact, according to the evidence given by the weights, the two models are almost equally supported by the data. Double BOOT offers a solution to this problem by performing a second layer of bootstrapping that uses a bootstrap data-generating process to weight each of the original candidate models according to their prevalence (measured through *w_i_*) as “best” models among the original *B* bootstrap replicate series. The logic used here could be extended to the case of many competing models where it seems reasonable to perform a second layer of bootstrapping based on how well each candidate model is supported by the data, rather than a single layer where the bootstrapping is based on a single model that essentially assumes full support from the available data. The difference in the double BOOT weights compared with the original BOOT weights would depend on a number of factors, including the number of candidate models that are “close” in terms of support offered by the data and the similarity of these models in terms of model structure. Irrespective of the change in the double BOOT weights, we believe the reweighting to be important—inherent to the success of the bootstrap is the premise that the data-generating process should mimic the true underlying process as closely as possible. In the case of something as complex as a model-selection process, the weights effectively measure a state of belief about the set of candidate models. Thus, our bootstrap resamples mimic that state of belief by generating data sets arising from a variety of candidate models in proportion to our confidence that such models are the correct ones.

The use of the bootstrap to tune another initial bootstrap algorithm has a long history. For example, Efron (1983) used a second level of bootstrap resampling to reduce the bias of the apparent error rate of a linear discriminant rule. Efron termed his method a “double bootstrap” because it involved a second layer of *B* resamples to bias correct an initial bootstrap bias-corrected estimate. Beran (1987) and Hall (1986) discussed the use of second-level resampling to correct for coverage error in confidence intervals. Hall and Martin (1988) proposed a general framework for bootstrap iteration for which the second-level resamples were used to estimate and correct for the error in the original bootstrap procedure. Loh (1987) also used a second layer of bootstrap resamples to correct confidence interval endpoints. However, the methods of Beran (1987), Hall (1986), and Loh (1987) differ in the way the bootstrap critical points are modified. In our approach, the first-layer bootstrap resamples are used to generate an initial set of weights for the set of candidate models. In one way, these weights can themselves be considered as outputs from the initial bootstrap procedure. But, of course, these weights are not “correct” because of the way the bootstrap resamples are constructed in the generalized linear model context. Because the resampling is based on model residuals, there is a tendency for the initial bootstrap step to favor (i.e., give higher weight to) the model from which the original residuals were obtained. Our second-layer bootstrap resampling is directed at addressing this problem, by using the information gleaned from the initial bootstrap step as a starting point to constructing second-level resamples based on residuals not from a single model fit, but rather from a weighted set of plausible candidate models. Our method is a fully frequentist analog of the bootstrap-after-Bayesian model averaging approach proposed by Buckland et al. (1997). In their paper, the authors had observed that a single-layer bootstrap model averaging approach tended to favor the initial model on which resamples were based. They suggested that an initial Bayesian model averaging (BMA) step could be used to provide a weighted set of models from which resamples could be based in a second bootstrap model selection step. Our method takes a fully frequentist approach by adopting bootstrap methods at both steps.

The form of BMA that will be used in our paper is based on AIC as described in Clyde (2000). In the context of Model [1], BMA based on AIC proceeds by assigning each candidate model *i* a posterior probability given by the following formula:


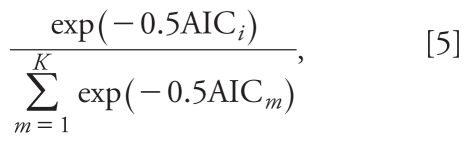


where AIC*_i_* is the AIC for candidate model *i* and *K* is the number of candidate models. The estimated mortality effect is obtained by weighting the PM effect estimates obtained from each model by its posterior probability.

In the context of our analyses, it is worth discussing the interpretation of the weighted average effect estimates obtained from BOOT, double BOOT, and BMA. These quantities, which are obtained by weighting estimates of the effect of an increase in PM_2.5_ on a single day’s mortality, may be viewed as weighted or model-averaged estimates of the effect of an increase in PM_2.5_ on a single day’s mortality. However, care should be taken when using model averaged estimates because the interpretation of particular parameters may change when other variables, such as copollutants, are added to the model (Lukacs et al. 2009; Thomas et al. 2007). Indeed, not all researchers would agree with the process of averaging estimates obtained using different lags of PM_2.5_. Some advocate that model averaging is best suited for making predictions (Thomas et al. 2007). In this regard, we also investigate the predictive performance of the three model-averaging procedures considered in this article.

## Results

We used the statistical package R along with packages “boot,” “splines,” and “tseries” for all the analyses (R Development Core Team 2009). Computational constraints meant that producing estimates of the standard errors (SEs) for values presented in Tables 1 and 2 was not feasible, and the provision of SEs for simulated values is not common practice in studies of this kind.

### Simulation study

We used the 730 days of data from Seattle, Washington, along with the specification of Model [1] to generate mortality time series where the effect of PM_2.5_ on mortality was known. Generating mortality time series was achieved by producing mortality counts on day *t* that were Poisson distributed with mean μ*_t_*


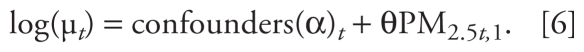


We considered three different specifications of confounders(α = 1.2)*_t_*

**Specification A**


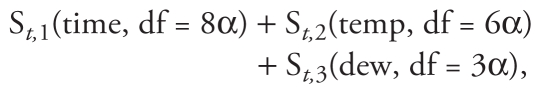


**Specification B**


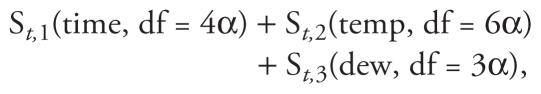


**Specification C**


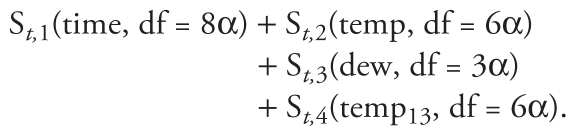


In the above equations, θ is the known PM_2.5_ effect, and temp, temp_13_, and dew represent the current day’s temperature, temperature of the previous 3 days, and current day’s dew point temperature, respectively. The functions S*_t_*_,_*_j_*() are smooth natural cubic spline functions with the indicated degrees of freedom. To ensure that the degrees of freedom take integer values, the values of 8α, 6α, 4α, and 3α are rounded to the nearest integer. To find realistic representations of the S*_t_*_,_*_j_*(), we fitted Model [6], using each specification of confounders(α = 1.2)*_t_* to the actual Seattle data using a Poisson log-linear generalized linear model with an offset term allowing the effect of PM_2.5_ to be set equal to θ. The offset term allows a term to be included in a generalized linear model with a known, rather than an estimated, coefficient value. We used the fitted values from these models to generate daily Poisson mortality estimates that incorporate a known PM_2.5_ effect θ. Three values of θ: 0, 0.0003, and 0.001 were considered.

To implement model averaging, a set of candidate models was required. We considered two sets of candidate models that were defined by Model [1] with α taking 10 equally spaced values ranging from α = 0.3 to α = 3, confounders(α)*_t_* as defined in specification A, and either three lags of PM_2.5_ (PM_2.5_*_t_*_,0_, PM_2.5_*_t_*_,1_, PM_2.5_*_t_*_,2_) or one lag of PM_2.5_ (PM_2.5_*_t_*_,1_). In the case of three lags of PM_2.5_, we have a set of 10 × 3 = 30 candidate models, and in the case of one lag of PM_2.5_, a set of 10 × 1 = 10 candidate models. Similar methods for defining the tuning parameter α for time and weather variables have been used in previous investigations (Dominici et al. 2004; Roberts 2004). The number of parameters estimated in each candidate model is equal to the total number of degrees of freedom used in the S*_t_*_,_*_j_*() plus 1 for the intercept and 1 for the estimated PM_2.5_ effect.

For mortality generated using the confounders(α = 1.2)*_t_* specification A, the “true” model is contained among both sets of candidate models, but for mortality generated using confounders(α = 1.2)*_t_* specifications B and C, this is not the case. In specification B, the degrees of freedom used for time have been halved for each value of α compared with the candidate models; whereas, specification C includes temp_13_, a variable that is not included in any of the candidate models. These latter two situations are perhaps more realistic because in practice no candidate model would correspond to the true model.

In the simulations, *B* = 1,000 was used in BOOT and for both layers of double BOOT. The simulations were conducted by generating sets of 1,000 mortality time series defined by Model [6] with α = 1.2, one of the confounder specifications A, B, or C, and θ and then by applying BOOT, double BOOT, and BMA using the two sets of candidate models [i.e., with 3 lags of PM_2.5_ (30 candidate models total) or 1 lag of PM_2.5_ (10 candidate models total)]. Table 1 contains the results of these simulations. In the simulations involving 30 candidate models, it is evident from the smaller root-mean-squared error (RMSE) values that double BOOT has superior performance to that of both BOOT and BMA. The breakdown of RMSE into bias and SE components shows that the improvement in performance offered by double BOOT is principally due to the lower SE of the estimates obtained by this method. In the simulations involving 10 candidate models, the methods offer similar performance.

For the simulations with 30 candidate models and confounders(α = 1.2)*_t_* = specification A, we investigated the use of standard AIC model selection (results not shown) by basing estimates on the single model selected as “best” based on AIC. Performance, as measured by RMSE, was substantially worse than that of double BOOT, BOOT, and BMA, with the average values of RMSE of approximately 1.90 for each of the three scenarios considered.

As a final comparison we compared the predictive performance of the three methods using both simulated and actual mortality data. For each mortality time series, we randomly removed 100 observations and applied BOOT, double BOOT, and BMA to the remaining data to obtain predictions for the removed observations. The predictions were computed as weighted averages of the predictions obtained from each candidate model weighted by the weight or probability assigned to that model. The performance of each method was based on the predictive mean squared error (PMSE) computed as {(*y*_1_ – &*ycirc;*_1_)^2^ +…+ (*y*_100_ – &*ycirc;*_100_)^2^}/100, where *y_i_* and &*ycirc;_i_* are the actual and predicted mortality estimates, respectively. For a given mortality time series, we repeated the process of randomly removing 100 data points and computing the PMSE 100 times. Table 2 reports the number of times (out of 100) that each method had a better predictive performance than alternative methods based on lower PMSE. It is clear that double BOOT has predictive performance superior to that of BOOT, with double BOOT having a smaller PMSE about 70% of the time. The results also provide support for double BOOT versus BMA, with double BOOT providing the same or better predictive performance in two of the three model-specific simulations and in three of the five city-specific simulations.

### Application

Tables 3 and 4 show the results of applying the three model-averaging methods and standard AIC to the five cities described above. We calculated the SE values in Table 3 using equation 4 of Burnham and Anderson (2004). For these five cities, the estimates obtained from the three model-averaging methods were similar and the conclusions drawn about the association between PM_2.5_ and mortality would be essentially the same. However, the results also illustrate that the estimates obtained from standard AIC can be significantly different to those obtained from model averaging. The SEs assigned to the estimates obtained from standard AIC are smaller because these SEs do not take into account the model selection process that was used to find the single best model.

The reason for the differences in the estimates obtained from the three model-averaging methods based on 30 candidate models compared with standard AIC is a result of the model-averaging methods assigning nonnegligible weights to a number of candidate models. Within each city, the three model-averaging techniques tended to assign nonnegligible weights to three models corresponding to the three different lags of PM_2.5_ but the same level of confounder adjustment α. Comparing the weights obtained from BOOT and double BOOT illustrates that the second bootstrap layer can result in substantial changes to the weights assigned to each model. For example, for Seattle and Tampa in some situations the weights assigned to candidate models differ by approximately 40%.

## Discussion

We have illustrated that double BOOT model averaging can offer benefits over BMA and BOOT for both estimation and prediction. The benefits were particularly noticeable for double BOOT compared with BOOT. This increased performance was attributable to a reduction in the variance of the estimates obtained from double BOOT compared with BOOT and BMA. An interesting observation was that the bias of the estimates obtained from double BOOT was larger than the estimates obtained from BOOT and BMA when the “true” model was contained among the candidate models. This was not the case, however, when the “true” model was not among the candidate models because the double BOOT procedure tended to give less weight to the true model as a consequence of the second bootstrap layer moving some of the weight from the true model to other plausible models. Of course, this phenomenon could not occur in the simulations where the “true” model was not among the candidate models, and the result was that double BOOT had slight improvements in terms of lower bias.

A report of particular relevance to the present study is that of Buckland et al. (1997) who investigated various forms of bootstrap model averaging, including the BOOT method in the present investigation. Buckland et al. (1997) and Claeskens and Hjort (2008) each provide excellent introductory treatments of the issues surrounding model selection and model averaging. Burnham and Anderson (2002) showed that AIC can be derived as a Bayesian result and that the AIC-based BMA weights used in the present paper correspond to posterior model probabilities. Unlike the implementation in this report, BMA can also be implemented by explicitly assigning prior model probabilities (Hoeting et al. 1999; Koop and Tole 2004). In the present setting, AIC-based BMA has the advantage of using objective prior distributions (Clyde 2000) and ease of implementation, compared with explicitly assigned prior model probabilities. An obvious disadvantage of AIC-based BMA is that is does not allow for the incorporation of prior information about the importance of a variable.

It is important to note that the use of BMA applied to time series studies of air pollution and mortality, and in particular the approach of Koop and Tole (2004), has received some criticism in the literature (Crainiceanu et al. 2008; Thomas et al. 2007). In this study we have attempted to avoid these same criticisms by ensuring that when illustrating our proposed averaging method we did so over a range of plausible candidate models, ensuring that a measure of air pollution exposure is included in each candidate model, focusing on single-pollutant models, and also investigating predictive performance. We are of the view that a carefully applied model-averaging procedure can provide useful insight into understanding air pollution health effects by, for example, providing information on how much the data support various models, helping practitioners to appreciate and allow for the effects of model selection and uncertainty, and in some circumstances providing improved estimators of air pollution health effects. However, we are also of the view that the use of model averaging does not negate the need for careful planning and data-gathering processes along with detailed investigations of models arising from a suitably rich set of initial covariates to find an initial and sufficiently rich plausible set of candidate models. We also believe that future comparisons of results obtained from model averaging with traditional methods such as standard AIC would prove valuable.

## Figures and Tables

**Table 1:** Results of simulations that compare the statistical properties of BOOT, double BOOT, and BMA for estimating the mortality effect of PM_2.5_.

	Method		
Specifications	BOOT	Double BOOT	BMA
No. of candidate models:^a^*K* = 30			
Mortality model:^b^ confounders(α = 1.2)*_t_* = specification A, and 1000θ = 0			

RMSE^c^	1.50	1.38	1.48
Bias/SE^d^	−0.28/1.47	−0.28/1.36	−0.25/1.46

Mortality model: confounders(α = 1.2)*_t_* = specification A and 1000θ = 0.3			
RMSE	1.54	1.43	1.51
Bias/SE	−0.38/1.49	−0.40/1.37	−0.35/1.47

Mortality model: confounders(α = 1.2)*_t_* = specification A and 1000θ = 1			
RMSE	1.47	1.39	1.44
Bias/SE	−0.42/1.41	−0.48/1.30	−0.39/1.39

Mortality model: confounders(α = 1.2)*_t_* = specification B and 1000θ = 0.3			
RMSE	1.36	1.25	1.34
Bias/SE	−0.07/1.36	−0.06/1.25	−0.07/1.34

Mortality model: confounders(α = 1.2)*_t_* = specification C and 1000θ = 0.3			
RMSE	1.50	1.38	1.48
Bias/SE	0.08/1.50	0.03/1.38	0.10/1.48

No. of candidate models: *K* = 10			
Mortality model: confounders(α = 1.2)*_t_* = specification A and 1000θ = 0.3			
RMSE	1.34	1.34	1.33
Bias/SE	−0.21/1.32	−0.23/1.32	−0.19/1.32

Mortality model: confounders(α = 1.2)*_t_* = specification B and 1000θ = 0.3			
RMSE	1.28	1.28	1.28
Bias/SE	−0.05/1.28	−0.04/1.28	−0.06/1.28

Mortality model: confounders(α = 1.2)*_t_* = specification C and 1000θ = 0.3			
RMSE	1.33	1.32	1.33
Bias/SE	0.17/1.32	0.15/1.32	0.18/1.32

a The number of candidate models used in the three model-averaging procedures.

b The specification of confounders(α = 1.2)*_t_* and θ used in Equation [6] to simulate mortality.

c 1,000 times the RMSE of the estimates of θ computed over 1,000 simulated mortality time series.

d 1,000 times the average bias and SE of the estimates of θ computed over 1,000 simulated mortality time series.

**Table 2:** Results of simulations comparing the predictive performance of BOOT, double BOOT, and BMA using 30 candidate models.

	Comparison^a^		
	Double BOOT vs. BMA	Double BOOT vs. BOOT	BMA vs. BOOT
Model^b^			
Confounders(α = 1.2)*_t_* = specification A and 1000θ = 0.3	49	71	66
Confounders(α = 1.2)*_t_* = specification B and 1000θ = 0.3	61	73	55
Confounders(α = 1.2)*_t_* = specification C and 1000θ = 0.3	51	69	58
City^c^			
Birmingham	63	78	50
Orlando	68	64	36
Seattle	72	47	23
St. Louis	24	88	83
Tampa	41	91	82

a Numbers indicate the number of simulations (out of 100 total) for which one method performed better than a comparison method based on lower PMSE estimates.

b The specification of confounders(α = 1.2)*_t_* and θ used in Equation [6] to simulate mortality.

c The city from which the actual mortality data corresponds.

**Table 3:** Results of applying BOOT, double BOOT, BMA, and standard AIC to five U.S. cities.^a^

	City				
Method	Birmingham	Orlando	Seattle	St. Louis	Tampa
No. of candidate models:^b^*K* = 30					
BOOT	0.50^c^ (1.55)^d^	0.17 (2.57)	−2.26 (1.42)	−1.11 (2.19)	3.01 (1.86)
Double BOOT	0.30 (1.57)	−0.08 (2.57)	−2.09 (1.43)	−0.92 (2.21)	2.93 (1.89)
BMA	0.42 (1.59)	−0.13 (2.58)	−2.19 (1.41)	−1.09 (2.26)	3.09 (1.84)
Standard AIC	1.29 (1.30)	1.69 (2.10)	−2.61 (1.34)	−1.92 (1.97)	3.22 (1.75)

No. of candidate models: *K* = 10					
BOOT	1.31 (1.30)	−1.52 (2.15)	−1.43 (1.31)	−0.86 (2.04)	3.26 (1.74)
Double BOOT	1.32 (1.30)	−1.52 (2.15)	−1.39 (1.32)	−0.85 (2.05)	3.31 (1.74)
BMA	1.34 (1.32)	−1.54 (2.15)	−1.45 (1.30)	−1.08 (2.14)	3.33 (1.74)
Standard AIC	1.29 (1.30)	−1.53 (2.15)	−1.51 (1.29)	−0.87 (2.01)	3.19 (1.75)

a The model specification is Model [6] with α = 1.2 and confounder specification A.

b The number of candidate models used in the three model-averaging procedures.

c 1,000 times the estimated mortality effect of PM_2.5_.

d 1,000 times the SE of the estimated mortality effect of PM_2.5_.

**Table 4:** Weight or posterior probability assigned to candidate models for data from five U.S. cities.

	Candidate model^a^				
City/method	Model 1	Model 2	Model 3	Model 4	Model 5
Birmingham	*j* = 0, α = 1.5	*j* = 1, α = 1.5	*j* = 2, α = 1.5		

Estimate^b^	−0.858	1.293	0.476		
BOOT^c^	22	36	23		
Double BOOT^c^	26	25	20		
BMA^c^	18	24	16		

Orlando	*j* = 0, α = 0.6	*j* = 1, α = 0.6	*j* = 2, α = 0.6		

Estimate	−0.730	−1.530	1.692		
BOOT	26	24	41		
Double BOOT	28	26	32		
BMA	25	31	33		

Seattle	*j* = 0, α = 0.9	*j* = 1, α = 0.9	*j* = 2, α = 0.9		

Estimate	−2.606	−1.506	−1.849		
BOOT	57	15	10		
Double BOOT	40	18	16		
BMA	51	15	22		

St. Louis	*j* = 0, α = 0.3	*j* = 1, α = 0.3	*j* = 2, α = 0.3	*j* = 2, α = 0.9	

Estimate	0.193	−0.872	−1.916	−2.201	
BOOT	22	23	44	1	
Double BOOT	27	24	33	2	
BMA	13	14	21	10	

Tampa	*j* = 0, α = 0.9	*j* = 0, α = 1.2	*j* = 1, α = 0.9	*j* = 1, α = 1.2	*j* = 2, α = 1.2

Estimate	3.341	3.219	3.532	3.192	1.345
BOOT	12	52	4	14	10
Double BOOT	16	32	9	18	12
BMA	15	21	20	21	5

Results are reported only for candidate models receiving a weight or probability ≥ 10%.

a The candidate model to which the weight or probability is assigned; *j* corresponds to the lag of PM_2.5_ and α indicates the degree of confounder adjustment for models with confounder(α)_t_ specification A.

b 1,000 times the estimated effect of PM_2.5_ obtained from the given candidate model.

c Weight or posterior probability assigned to each candidate model.
